# Retinal structural and microvascular changes in myelin oligodendrocyte glycoprotein antibody disease and neuromyelitis optica spectrum disorder: An OCT/OCTA study

**DOI:** 10.3389/fimmu.2023.1029124

**Published:** 2023-01-30

**Authors:** Yanlin Lang, William Robert Kwapong, Lingyao Kong, Ziyan Shi, Xiaofei Wang, Qin Du, Bo Wu, Hongyu Zhou

**Affiliations:** Department of Neurology, West China Hospital, Sichuan University, Chengdu, China

**Keywords:** neuromyelitis optica spectrum disorder, myelin oligodendrocyte glycoprotein antibody disease, optical coherence tomography, optical coherence tomography angiography, macula microvasculature

## Abstract

**Purpose:**

To compare the optical coherence tomography (OCT)/OCT angiography (OCTA) measures in patients with neuromyelitis optica spectrum disorder (NMOSD) and myelin oligodendrocyte glycoprotein antibody disease (MOGAD).

**Methods:**

Twenty-one MOG, 21 NMOSD, and 22 controls were enrolled in our study. The retinal structure [retinal nerve fiber layer (RNFL) and ganglion cell–inner plexiform layer (GCIPL)] was imaged and assessed with the OCT; OCTA was used to image the macula microvasculature [superficial vascular plexus (SVP), intermediate capillary plexus (ICP), and deep capillary plexus (DCP)]. Clinical information such as disease duration, visual acuity, and frequency of optic neuritis and disability was recorded for all patients.

**Results:**

Compared with NMOSD patients, MOGAD patients showed significantly reduced SVP density (*P* = 0.023). No significant difference (*P* > 0.05) was seen in the microvasculature and structure when NMOSD-ON was compared with MOG-ON. In NMOSD patients, EDSS, disease duration, reduced visual acuity, and frequency of ON significantly correlated (*P* < 0.05) with SVP and ICP densities; in MOGAD patients, SVP correlated with EDSS, duration, reduced visual acuity, and frequency of ON (*P* < 0.05), while DCP density correlated with disease duration, visual acuity, and frequency of ON.

**Conclusions:**

Distinct structural and microvascular changes were identified in MOGAD patients compared with NMOSD patients suggesting that the pathological mechanisms are different in NMOSD and MOGAD. Retinal imaging *via* the SS-OCT/OCTA might have the potential to be used as a clinical tool to evaluate the clinical features associated with NMOSD and MOGAD.

## Introduction

Myelin oligodendrocyte glycoprotein (MOG) is a glycoprotein expressed on oligodendrocytes and is a minor component forming the myelin sheath in the central nervous system (1). The immune attack in MOG antibody disease (MOGAD) is associated with myelin and oligodendrocyte damage, resulting in heterogeneous clinical manifestations such as optic neuritis (ON), reduced/loss of vision, myelitis, seizures, brainstem syndromes, and encephalitis (2–4). To date, it is suggested that clinical manifestations in MOGAD differ from multiple sclerosis (MS) (5) but overlap with aquaporin-4 antibody seropositive neuromyelitis optica spectrum disorder (NMOSD AQP4+) (6, 7); optic neuritis and acute myelitis in MOGAD have similarities with clinical manifestations of NMOSD and can only be distinguished by detecting MOG antibodies. Even though testing for MOG antibodies can help differentiate these disorders, MOG testing is time-consuming and not available in most countries; moreover, albeit testing of MOG antibodies is very precise (8), false positives can still arise due to its low prevalence worldwide even in individuals with demyelinating disorders with ON (9, 10). MOGAD and NMOSD have different pathological mechanisms and different target cell damage. Given the different clinical outcomes and treatment strategies between NMOSD and MOGAD, accurate monitoring and evaluation would facilitate optimal treatment decisions and prognosis prediction. Thus, it would be supportive to have other modalities that may help in the quick and early identification of patients with MOGAD and help differentiate it from NMOSD.

Accumulating reports (10, 11) suggested that MOGAD with ON has recurrent and wide-range optic disc edema at the beginning of the disease cascade when compared with NMOSD; these optic nerve head changes cause severe neuroaxonal and microvascular impairment in the retina. Optical coherence tomography (OCT)/OCT angiography (OCTA) can quantify the retinal structural thickness and microvascular density changes and may serve as a reliable tool to differentiate MOGAD from NMOSD.

In this observational cross-sectional study, we utilized the swept-source OCT (SS-OCT) and SS-OCTA to characterize the retinal structural and microvascular changes in NMOSD and MOGAD patients when compared with controls; we also explored the association between OCT/OCTA parameters in MOGAD and NMOSD with their disease duration, disability, and visual acuity.

## Methods

This exploratory cross-sectional observational study was approved by the Institutional Review Board of West China Hospital, Sichuan, China, and all participants provided written informed consent before enrolling in our study.

**Figure 1:**
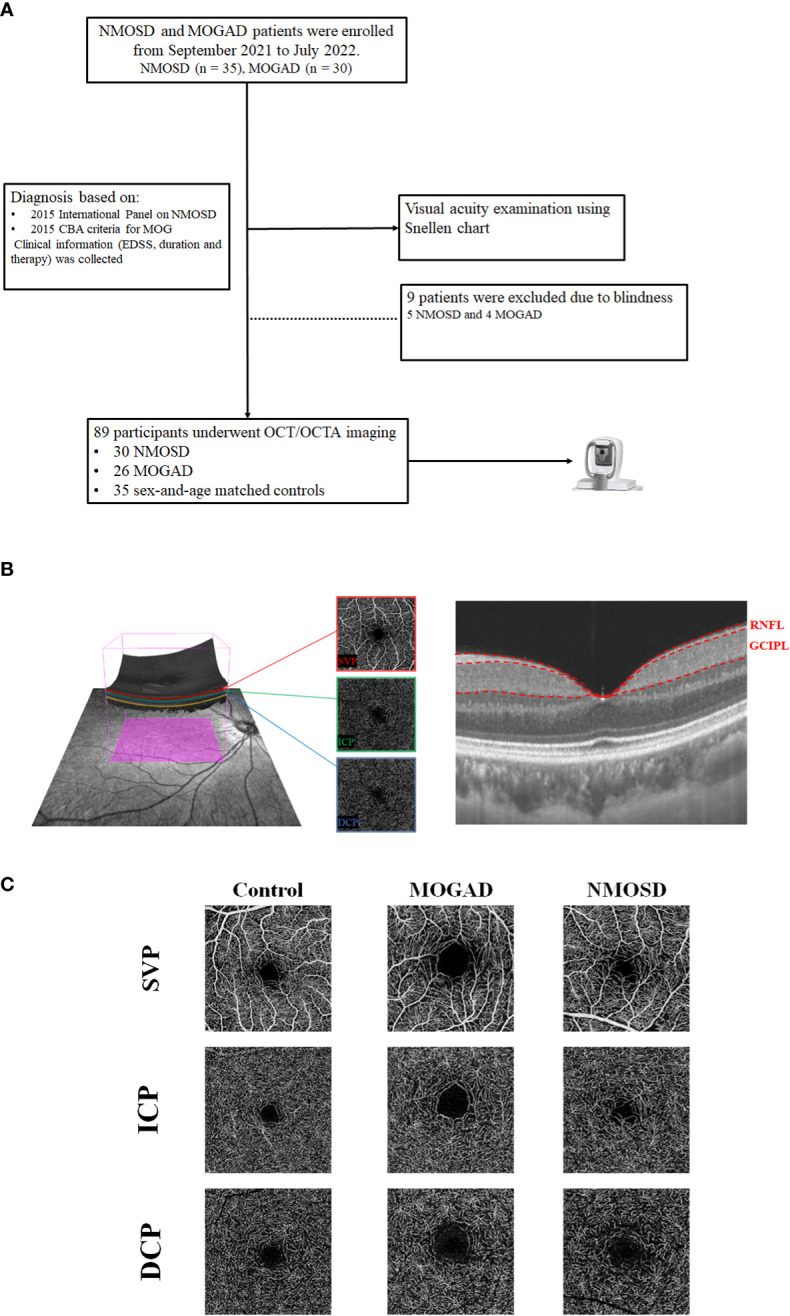
The flowchart of our participants and segmentation of OCT/OCTA images. **(A)** The flowchart of the participants in our study. **(B)** The segmentation of three macular microvascular plexuses and the retinal thickness of RNFL and GCIPL in an area of 3 × 3 mm around the fovea. SVP was defined as the microvasculature between the base of the retinal nerve fiber layer (RNFL) to the junction between the inner plexiform layer (IPL) and inner nuclear layer (INL); ICP was the microvessels between the IPL/INL junction to the INL/outer plexiform layer (OPL) junction, while DCP was the microvessels between the INL/OPL junction to 25 µm below the OPL. **(C)** The en face angiograms of our participants.

Twenty-two MOGAD and 23 NMOSD patients were recruited from the Neurology Department of West China Hospital. NMOSD patients tested seropositive by cell-based assay (EUROIMMUN AG, Luebeck, Germany) as previously reported (12), and the diagnosis was based on the 2015 International Panel on NMOSD (13), while MOGAD patients tested seropositive by the cell-based assay as previously reported (14) (**Figure 1A**). Patients with ON less than 6 months before evaluation were excluded from our study. The inclusion criteria for MOGAD and NMOSD patients were as follows: 1) no sudden vision loss or eyeball pain occurred in the past 6 months, 2) long-term follow-up in our hospital with complete data and without a history of seizures, and 3) patients who could cooperate with our study and signed informed consent. The exclusion criteria were as follows: 1) possibly confounding neurological or ophthalmological disorders, 2) eyes with prior ocular surgery or trauma or acute optic neuritis within the preceding 6 months, 3) refractive error of ±6 D (diopters), and 4) inability to cooperate with our study. Clinical variables such as frequency of optic neuritis, visual acuity under illumination, and Expanded Disability Status Scale (EDSS) were assessed and recorded.

For comparison, 22 healthy controls with no history of neurological or neuropsychological diseases were included in our study. Participants with uncontrolled hypertension or diabetes, a history of ocular surgery, glaucoma, and other ophthalmologic diseases were also excluded from our study for all groups.

All participants underwent comprehensive visual acuity using the Snellen chart. Each participant’s visual acuity for both eyes was obtained under light and later converted to a logarithm of the minimum angle of resolution (LogMAR).

### SS-OCT/SS-OCTA imaging and examination

The SS-OCT/SS-OCTA tool (VG200S; SVision Imaging, Henan, China; version 2.1.016) was used to image the retinal structure and microvasculature for all participants. The specification of this OCT/OCTA tool is well documented in previous reports (15–17). Imaging of the retinal structure was performed with 18 radial B-scans positioned on the fovea. Automatic segmentation of the retinal thickness was done by the OCT tool. Our current study assessed the macular retinal nerve fiber layer (mRNFL) and ganglion cell–inner plexiform layer (GCIPL) in a 3 × 3-mm^2^ area around the fovea as shown in **Figure 1B**. The average thicknesses (measured in µm) of the retinal structure were obtained from the OCT tool.

The OCTA images covered an area of 3 × 3 mm^2^ around the fovea. The en-face angiograms of the superficial vascular complex (SVC) and deep vascular complex (DVC) were generated by the OCTA tool. The partition of the SVC and DVC slabs was set in the inner two-thirds and outer one-third border of GCIPL as shown in **Figure 1B**. The average percentages (%) of the microvasculature in the SVC and DVC were obtained from the OCTA tool.

The OCT/OCTA data displayed in our study followed the OSCAR-IB quality criteria (18) and APOSTEL recommendation (19).

### Statistical analyses

The Shapiro–Wilk test was used to test for the normality of our data. Continuous variables with normal distribution were expressed as mean ± standard deviation (SD), while skewed distribution was expressed as medians and interquartile ranges. Categorical variables were presented as frequencies and percentages. SS-OCT/SS-OCTA parameters among the groups were assessed using generalized estimating equations (GEEs) while adjusting for age and gender. The association between SS-OCT/SS-OCTA parameters and the clinical features was performed with GEE while adjusting for risk factors. A comparison between SS-OCTA parameters stratified by history of ON between MOGAD and NMOSD was performed using a linear mixed model while adjusting for risk factors and intereye dependencies. All analyses were performed with SPSS (version 26, SPSS Inc., Chicago, IL, USA). *P*-values less than 0.05 were considered statistically significant. This was an exploratory study, so no adjustment for multiple comparisons was made.

## Results

Our final data analyses included 40 eyes from 21 MOGAD patients (mean age = 33.67 ± 11.07 years), 42 eyes from 21 NMOSD patients (mean age = 33.76 ± 11.07 years), and 44 eyes from 22 healthy controls (mean age = 34.18 ± 10.88 years). Age and sex did not differ among the three groups (*P* > 0.05, **Table 1**). Thirteen MOGAD (61.9%; mean = 1 ON episode) and 11 NMOSD (52.4%; mean = 1 ON episode) patients had a history of ON. **Table 1** displays the demographics and clinical information of our participants.

**Table 1:** Demographic overview.

	HC	MOGAD	NMOSD
Participants (*N*)	22	21	21
Number of eyes (*N*)	44	40	42
Age (years, mean ± SD)	34.18 ± 10.88	33.67 ± 11.07	33.76 ± 11.07
Gender [female, *N* (%)]	17 (77.3)	12 (57.1)	18 (85.7)
EDSS (median, IQR)	–	1.0 (0 - 2.25)	1.5 (1.0 - 3.0)
Patients with a history of ON [*N* (%)]	–	13 (61.9%)	11 (52.4%)
Number of ON episodes (median, range)	–	1.0 (0 - 5)	1.0 (0 - 5)
Disease duration (years, mean ± SD)	–	2.57 ± 2.66	3.17 ± 2.60
VA (LogMAR, mean ± SD)	−0.05 ± 0.10	0.03 ± 0.10	0.18 ± 0.41
Immunotherapy			
None (%)	–	5 (23.8%)	1 (4.8%)
Prednisone (%)	–	7 (33.3%)	1 (4.8%)
Mycophenolate mofetil (%)	–	7 (33.3%)	12 (57.1%)
Azathioprine (%)	–	1 (4.8%)	2 (9.5%)
Rituximab (%)	–	1 (4.8%)	5 (23.8%)

EDSS, Expanded Disability Status Scale; VA, visual acuity; HC, healthy control; MOGAD, myelin oligodendrocyte glycoprotein antibody disease; NMOSD, neuromyelitis optica spectrum disorder; N, number of subjects; ON, optic neuritis.

### Comparison of SS-OCT/SS-OCTA measures among the groups

**Figure 2:**
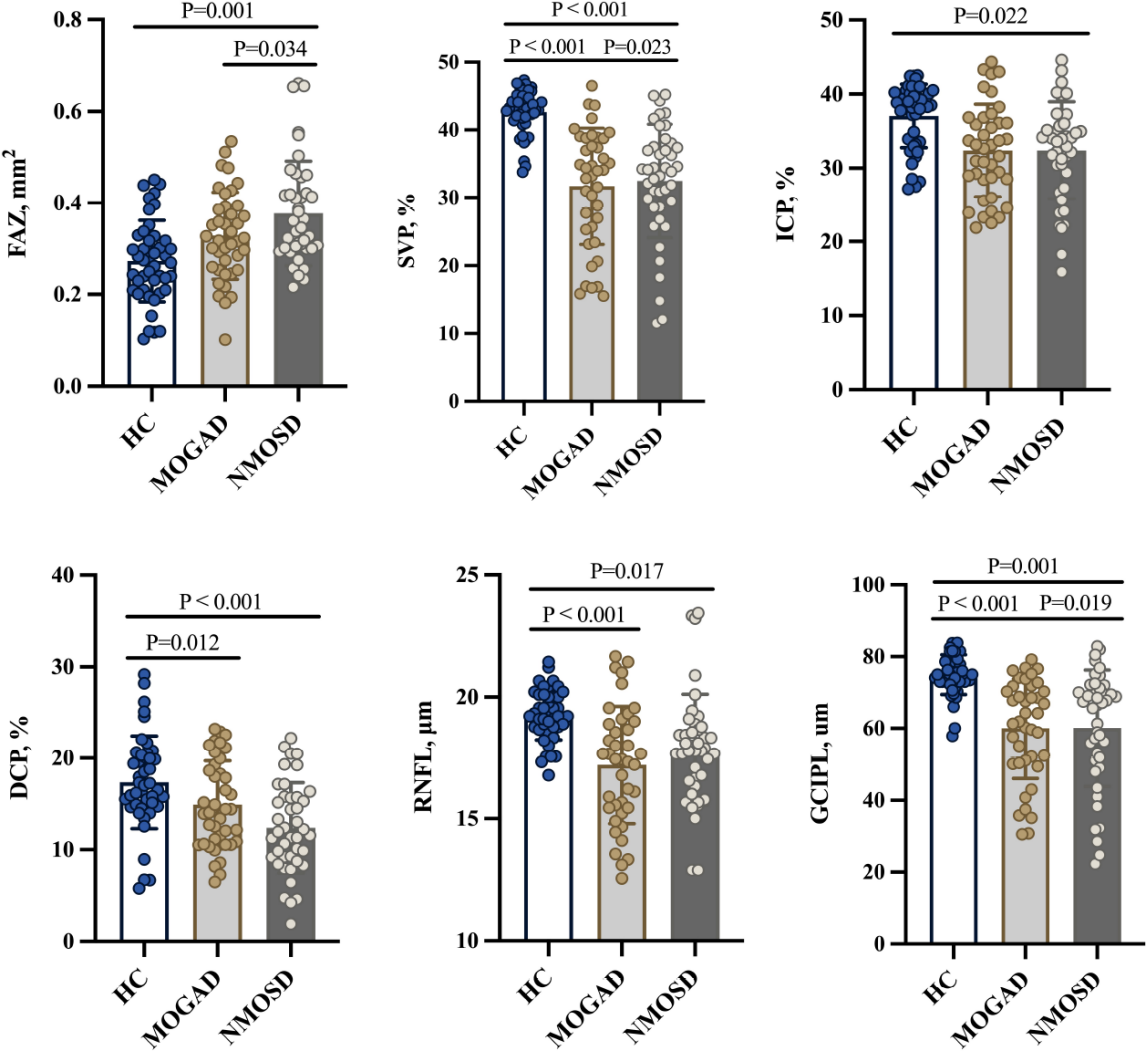
Comparison of OCT/OCTA parameters. FAZ, foveal avascular zone; SVP, superficial vascular plexus; ICP, intermediate capillary plexus; DCP, deep capillary plexus; RNFL, retinal nerve fiber layer; GCIPL, ganglion cell and inner plexiform layer; HC, healthy control; MOGAD, myelin oligodendrocyte glycoprotein antibody disease; NMOSD, neuromyelitis optica spectrum disorder.

MOGAD and NMOSD patients showed thinner RNFL and GCIPL thicknesses (*P* < 0.05, **Table 2** and **Figure 2**) when compared with controls; compared with NMOSD patients, MOGAD patients had thinner GCIPL thickness (*P* = 0.019, **Table 2** and **Figure 2**).

**Table 2:** Comparison of OCT/OCTA parameters.

	HC	MOGAD	NMOSD	*P* (MOGAD vs. HC)	*P* (MOGAD vs. NMOSD)	*P* (NMOSD vs. HC)
FAZ, mm^2^	0.273 ± 0.089	0.328 ± 0.095	0.377 ± 0.115	0.135	0.034*	0.001*
SVP, %	42.636 ± 3.195	31.705 ± 8.576	32.495 ± 8.359	<0.001*	0.023*	<0.001*
ICP, %	37.048 ± 4.288	32.376 ± 6.253	32.413 ± 6.582	0.172	0.304	0.022*
DCP, %	17.361 ± 5.091	14.911 ± 4.813	12.379 ± 4.924	0.012*	0.558	<0.001*
RNFL, μm	19.285 ± 1.039	17.206 ± 2.412	18.270 ± 3.582	<0.001*	0.057	0.017*
GCIPL, μm	74.985 ± 5.543	60.006 ± 13.880	60.087 ± 16.205	<0.001*	0.019*	0.001*

FAZ, foveal avascular zone; SVP, superficial vascular plexus; ICP, intermediate capillary plexus; DCP, deep capillary plexus; RNFL, retinal nerve fiber layer; GCIPL, ganglion cell and inner plexiform layer; HC, healthy control; MOGAD, myelin oligodendrocyte glycoprotein antibody disease; NMOSD, neuromyelitis optica spectrum disorder.

*P < 0.05.

**Figure 1C** shows the angiograms of our participants. Compared with controls, MOGAD patients showed reduced SVP (*P* < 0.001, **Table 2** and **Figure 2**) and DCP (*P* = 0.012, **Table 2** and **Figure 2**) densities. Similarly, NMOSD patients showed reduced SVP (*P* < 0.001, **Table 2** and **Figure 2**), ICP (*P* = 0.022, **Table 2** and **Figure 2**), and DCP (*P* < 0.001, **Table 2** and **Figure 2**) densities and enlarged FAZ area (*P* = 0.001, **Table 2** and **Figure 2**) when compared with controls.

MOGAD patients showed reduced SVP density (*P* = 0.023, **Table 2**) and thinner GCIPL thickness (*P* = 0.019, **Table 2**) when compared with NMOSD patients; the FAZ area was larger (*P* = 0.034) in NMOSD patients than in MOGAD patients.

**Supplementary Figure 1** shows the correlation between OCT and OCTA measures in both MOGAD and NMOSD patients. **Supplementary Figure 1A** shows the correlation in MOGAD patients, while **Supplementary Figure 1B** shows the correlation in NMOSD patients.

**Table 3** shows the SS-OCTA measures among the groups stratified by history of ON. Compared with controls, MOG-ON eyes showed reduced SVP (*P* < 0.001, **Table 3**) and ICP (*P* < 0.001, **Table 3**) densities and enlarged FAZ area (*P* = 0.02, **Table 3**), while MOG-NON eyes showed reduced SVP (*P* < 0.001, **Table 3**) and DCP (*P* < 0.001, **Table 3**) densities. MOG-ON eyes showed significantly reduced (*P* < 0.05, **Table 3**) SVP, ICP, and DCP densities when compared with MOG-NON eyes. NMOSD-ON eyes and NMOSD-NON eyes showed reduced microvascular densities and thinner retinal thicknesses when compared with controls. Importantly, NMOSD-ON eyes showed an enlarged FAZ area (*P* = 0.036, **Table 3**) when compared with MOG-ON eyes; NMOSD-NON eyes showed significantly reduced DCP density (*P* = 0.048, **Table 3**) when compared with MOG-NON eyes.

**Table 3:** OCTA results in MOGAD and NMOSD patients stratified by history of ON.

	MOG-ON	MOG-NON	NMOSD-ON	NMOSD-NON	*P* MOG-ON vs. MOG-NON	*P* MOG-ON vs. HC	*P* MOG-NON vs. HC	*P* MOG-ON vs. NMOSD-ON	*P* MOG-NON vs. NMOSD-NON	*P* NMOSD-ON vs. HC	*P* NMOSD-NON vs. HC
Number of eyes	18	22	18	24							
FAZ, mm^2^	0.336 ± 0.098	0.322 ± 0.095	0.420 ± 0.132	0.345 ± 0.090	0.862	0.020*	0.065	0.036*	0.829	<0.001*	0.001*
SVP, %	25.727 ± 6.996	36.597 ± 6.424	26.878 ± 8.667	36.707 ± 5.084	<0.001*	<0.001*	<0.001*	0.892	0.620	<0.001*	<0.001*
ICP, %	29.202 ± 5.817	34.972 ± 5.434	28.179 ± 7.113	35.589 ± 3.906	0.001*	<0.001*	0.495	0.054	0.626	<0.001*	0.008*
DCP, %	18.696 ± 3.892	11.814 ± 2.905	15.585 ± 5.043	9.972 ± 3.214	<0.001*	0.170	<0.001*	0.054	0.048*	0.139	<0.001*

FAZ, foveal avascular zone; SVP, superficial vascular plexus; ICP, intermediate capillary plexus; DCP, deep capillary plexus; HC, healthy control; MOGAD, myelin oligodendrocyte glycoprotein antibody disease; NMOSD, neuromyelitis optica spectrum disorder.

*P < 0.05.

**Figure 3:**
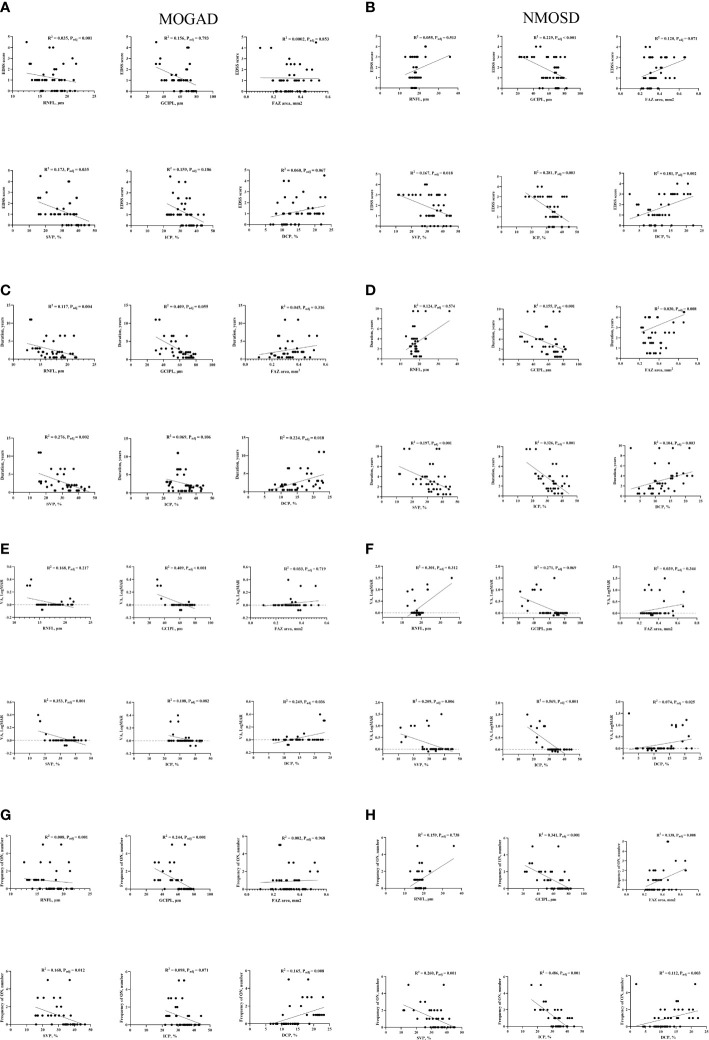
Correlation between clinical features and OCT/OCTA parameters in MOGAD and NMOSD patients. EDSS, Expanded Disability Status Scale; VA, visual acuity; FAZ, foveal avascular zone; SVP, superficial vascular plexus; ICP, intermediate capillary plexus; DCP, deep capillary plexus; RNFL, retinal nerve fiber layer; GCIPL, ganglion cell, and inner plexiform layer; HC, healthy control; MOGAD, myelin oligodendrocyte glycoprotein antibody disease; NMOSD, neuromyelitis optica spectrum disorder. Association of retinal parameters and EDSS **(A)**, Duration **(C)**, VA **(E)** and Frequency of ON **(G)** in MOGAD patients. Association of retinal parameters and EDSS **(B)**, Duration **(D)**, VA **(F)** and Frequency of ON **(H)** in NMOSD patients.

**Figure 3** shows the association between SS-OCT/SS-OCTA measures in MOGAD and NMOSD patients and their clinical features. RNFL thickness was significantly correlated with EDSS (*P* < 0.001) (**Figure 3A**), disease duration (*P* = 0.004) (**Figure 3C**), and frequency of ON (*P* = 0.001) (**Figure 3G**), while GCIPL correlated with reduced visual acuity (*P* < 0.001) (**Figure 3E**) and frequency of ON (*P* = 0.001) (**Figure 3G**) in MOGAD patients. GCIPL thickness in NMOSD patients significantly correlated with EDSS (*P* < 0.001) (**Figure 3B**), disease duration (*P* < 0.001) (**Figure 3D**), and frequency of ON (*P* < 0.001) (**Figure 3H**).

**Supplementary Figure 2** shows the correlation between OCT/OCTA measures and clinical features in the eyes of patients with MOGAD-ON and the eyes of patients with NMOSD-ON. **Supplementary Figures 2A, B** show the correlation between OCT/OCTA measures with visual acuity (measured in LogMAR) and frequency of ON in MOGAD-ON, while **Supplementary Figures 2C, D** show the correlation between OCT/OCTA measures with visual acuity (measured in LogMAR) and frequency of ON in NMOSD-ON.

In MOGAD patients, EDSS correlated (*P* = 0.035) with SVP, disease duration correlated with SVP (*P* = 0.002) and DCP (*P* = 0.018), and reduced visual acuity and frequency of ON significantly correlated (*P* < 0.05) with SVP and DCP, respectively, as shown in **Figure 3**. In NMOSD patients, EDSS, disease duration, reduced visual acuity, and frequency of ON correlated (*P* < 0.05, **Figure 3**) with retinal microvascular changes in the three plexuses.

## Discussion

Our current report showed retinal thinning and reduced microvascular densities in NMOSD and MOGAD patients compared with controls which are congruent with previous OCT/OCTA reports (20–23) indicating that neurodegeneration and microvascular impairment occur during the disease cascade. Our study showed thinner GCIPL thickness and reduced SVP density in MOGAD patients compared with NMOSD patients. In MOGAD patients, SVP density correlated with EDSS, disease duration, frequency of ON, and visual acuity, while DCP correlated with disease duration, visual acuity, and frequency of ON; RNFL thickness in MOGAD patients correlated with EDSS, disease duration, and frequency of ON. In NMOSD patients, SVP and ICP densities correlated with EDSS, disease duration, visual acuity, and frequency of ON, while GCIPL thickness correlated with EDSS, disease duration, and frequency of ON.

The novel findings in our study were significantly thinner GCIPL thickness and reduced SVP density in MOGAD compared with NMOSD. The SVP, located in the GCIPL, is responsible for the metabolic supply of neurons in these layers (24), where reduced thicknesses have been reported in both MOGAD and NMOSD (11, 22, 23). With regard to retinal neurodegeneration, it is suggested that retinal thinning in NMOSD is as severe as in MOGAD (25); similarly, a recent report showed that microvascular impairment in MOGAD and NMOSD was similar (23). However, a recent study showed that retinal thinning was more severe in MOGAD than in NMOSD which is congruent with our structural report (26). Our findings of thinner GCIPL thickness and reduced SVP density in MOGAD compared with NMOSD patients included patients with a history of optic neuritis. ON in MOGAD is often bilateral and characterized by retinal edema (27). During retinal edema, it suggested that GCIPL experiences severe neurodegeneration during the ensuing months (22, 27, 28). This structural thinning amasses with each added ON episode which occurs recurrently in MOGAD (25, 29). Although a single episode of ON does not lead to a devastating impairment (26), the highly frequent ON attacks accrue with GCIPL thinning. This is analogous to NMOSD, which is characterized by less frequent ON episodes. Due to the highly recurrent ON attacks in MOGAD, GCIPL thinning and reduced SVP density compared with NMOSD may reflect severe neurodegeneration and microvascular impairment.

In line with previous reports (30, 31), we found enlarged FAZ area as a unique characteristic in NMOSD patients but not in MOGAD patients irrespective of ON. We also showed that NMOSD eyes without ON showed reduced microvascular densities compared with controls. This development may be linked with the pathology of AQP4 antibodies since the parafoveal areas of the retina comprise the highest density of astrocytic Muller cells, which express AQP4 and have shown to be the targets of anti-AQP4 antibodies in NMOSD (32, 33). Enlarged FAZ area and reduced microvascular densities in NMOSD eyes without ON suggest that some activities occur during the subclinical phase in NMOSD and may initiate relapse-independent disease progression.

Concerning microvascular changes after ON, our current study did not find any significant difference between NMOSD-ON and MOG-ON patients which is in line with a previous report (23). It is suggested that ON in MOGAD is acute and severe which is similar to ON in NMOSD; however, the long-term prognosis is better in MOGAD compared with NMOSD. Thus, it is plausible to suggest that microvascular impairment after ON in MOGAD patients may be as severe as in NMOSD patients.

Although NMOSD and MOGAD patients presented with similar clinical features, dissimilar clinical associations were shown in MOGAD and NMOSD which may suggest different pathological processes and underlying macular structural and microvascular impairment. In NMOSD, reduced macula microvascular densities and GCIPL thinning significantly correlated with clinical disability, implying that the potential force of underlying neurodegeneration and microvascular impairment may lead to clinical disability measured by EDSS (34). Moreover, reduced macula microvascular densities and GCIPL thinning significantly correlated with disease duration and frequency of ON, while reduced microvascular densities significantly correlated with reduced visual acuity, which is consistent with OCT/OCTA reports (34–36). These findings were different from those in MOGAD, where clinical correlations were most identified with the SVP and DCP of the macula suggesting the pathological mechanisms are different in NMOSD and MOGAD. The differential association pattern demonstrated indicates that retinal imaging markers might have the potential to be used as a clinical tool to evaluate the clinical features associated with NMOSD and MOGAD.

We would like to acknowledge some limitations in our study. The exploratory and cross-sectional study design of the study limits the understanding of the results concerning the cause and effect. Secondly, our study focused on quantitative retinal microvasculature, while other central nervous system tissues such as the brain, spinal cord, and optic nerve were not evaluated; further studies with a comprehensive assessment of the central nervous system in both NMOSD and especially MOGAD are needed. Thirdly, the clinical relevance of macula microvasculature was assessed with clinical disability (EDSS), visual acuity, disease duration, and frequency of optic neuritis; further study is warranted to assess its value to cognition and treatment response. The data from our study were obtained from our hospital cohort which limits the generalizability of the data to the general population. There was a possibility of a selection bias caused by the exclusion of individuals with retinal abnormalities (high myopia) and unmatched gender among groups. This may have led to an underestimation of the observed correlations.

In conclusion, we showed that GCIPL thinning and reduced SVP density are severe in MOGAD than in NMOSD. We also showed that microvascular changes after ON in NMOSD may be as severe as in MOGAD. Importantly, we showed that enlarged FAZ area and reduced microvascular densities occur as unique features during the subclinical phase of NMOSD but not during MOGAD. Taken together, our study suggests that the OCT/OCTA tool can help facilitate the differentiation of MOGAD from NMOSD in settings with overlapping clinical features.

## Data availability statement

The original contributions presented in the study are included in the article/**Supplementary Material**. Further inquiries can be directed to the corresponding authors.

## Ethics statement

The studies involving human participants were reviewed and approved by the Institutional Review Board of West China Hospital, Sichuan, China. The patients/participants provided their written informed consent to participate in this study.

## Author contributions

YL: methodology, data curation, formal analysis, writing—original draft, and writing—review and editing. WK: methodology, data curation, formal analysis, writing—original draft, and writing—review and editing. LK: investigation and data curation. ZS: investigation and formal analysis. XW: investigation and formal analysis. QD: investigation and data curation. BW: conceptualization, methodology, validation, writing—review and editing, and supervision. HZ: conceptualization, methodology, validation, funding acquisition, writing—review and editing, and supervision. All authors contributed to the article and approved the submitted version.
