# Supplementary material for: Retinal structural and microvascular changes in myelin oligodendrocyte glycoprotein antibody disease and neuromyelitis optica spectrum disorder: An OCT/OCTA study
Source: Front Immunol. 2023 Jan 30;14:1029124. doi: 10.3389/fimmu.2023.1029124 (PMC9923098; doi:10.3389/fimmu.2023.1029124)
Supplement: Supplementary file 1 [file DataSheet_1.docx]

Supplementary figure 1

**Fig. S1. Correlation between OCTA measures and RNFL/GCIPL thickness in MOGAD (****Fig. S1A) and NMOSD (****Fig. S1B).** RNFL thickness significantly correlated with SVP (P < 0.01, Fig. S1A) and DCP (P＜0.01, Fig. S1A) while GCIPL correlated with SVP (P < 0.01, Fig. S1A), ICP (P < 0.01, Fig. S1A) and DCP (P＜0.01, Fig. S1A) in MOGAD patients. RNFL thickness in NMOSD patients significantly correlated with FAZ (P = 0.03) and SVP (P < 0.05) respectively in Fig. S1B. In NMOSD, GCIPL thickness correlated with FAZ(P＜0.01), SVP (P＜0.01), ICP (P＜0.01), and DCP (P＜0.01) respectively in Fig. S1B.

EDSS, Expanded Disability Standard Scale; VA, visual acuity; FAZ, foveal avascular zone; SVP, superficial vascular plexus; ICP, intermediate capillary plexus; DCP, deep capillary plexus; RNFL, retinal nerve fiber layer; GCIPL, ganglion cell, and inner plexiform layer; HC, healthy control; MOGAD, myelin oligodendrocyte glycoprotein antibody disease; NMOSD, neuromyelitis optica spectrum disorders

Supplementary figure 2

Fig. S2. Correlation between visual acuity/frequency of ON and OCT/OCTA parameters in MOGAD-ON eyes (Fig. S2 AB) and NMOSD-ON (Fig. S2 CD) eyes. VA correlated with RNFL (P = 0.032, Fig. S2A), GCIPL (P < 0.01, Fig. S2A) and SVP (P = 0.01, Fig. S2A) in MOGAD-ON eyes. VA and frequency of ON correlated with ICP (P＜0.001, Fig S2C; P=0.003, Fig S2D) in NMOSD-ON eyes.

VA, visual acuity; FAZ, foveal avascular zone; SVP, superficial vascular plexus; ICP, intermediate capillary plexus; DCP, deep capillary plexus; RNFL, retinal nerve fiber layer; GCIPL, ganglion cell, and inner plexiform layer; ON, optic neuritis; MOGAD, myelin oligodendrocyte glycoprotein antibody disease; NMOSD, neuromyelitis optica spectrum disorders
